# The Impact of Human-Robot Collaboration Levels on Postural Stability During Working Tasks Performed While Standing: Experimental Study

**DOI:** 10.2196/64892

**Published:** 2025-02-27

**Authors:** Daniele Bibbo, Giovanni Corvini, Maurizio Schmid, Simone Ranaldi, Silvia Conforto

**Affiliations:** 1 Department of Industrial, Electronic and Mechanical Engineering, Roma Tre University, Via Vito Volterra, 62 - Corpo B, Roma, 00146, Italy, 39 0657337298

**Keywords:** human-robot collaboration, cobot assistance, postural control, biomechanical risk, ergonomics, collaborative robot

## Abstract

**Background:**

The integration of collaborative robots (cobots) in industrial settings has the potential to enhance worker safety and efficiency by improving postural control and reducing biomechanical risk. Understanding the specific impacts of varying levels of human-robot collaboration on these factors is crucial for optimizing cobot use.

**Objective:**

This study aims to investigate the biomechanical effects of different levels of human-robot collaboration on postural stability and control during simulated working tasks.

**Methods:**

A total of 14 participants performed simulated cashier working activities under 4 different collaboration modalities, with increasing levels of cobot assistance: full (Fu), half robot touch (HRT), half robot (HRb), and full robot (FRb). Center of pressure trajectories were extracted from 2 force plates’ data to calculate 4 posturography parameters—mean distance (MDIST), mean velocity (MVELO), 95% confidence ellipse area (AREA-CE), and sway area (AREA-SW)—which were analyzed to assess the impact of cobot intervention on postural control.

**Results:**

Nonparametric tests showed significance in the effect of the collaboration modalities on the 4 analyzed parameters. Post hoc tests revealed that FRb modality led to the greatest enhancement in postural stability, with a reduction in MDIST (4.2, SD 1.3 cm in Fu vs 1.6, SD 0.5 cm in FRb) and MVELO (16.3, SD 5.2 cm/s in Fu vs 7.9, SD 1.1 cm/s in FRb). AREA-CE and AREA-SW also decreased significantly with higher levels of cobot assistance (AREA-CE: 134, SD 91 cm² in Fu vs 22, SD 12 cm² in FRb; AREA-SW: 16.2, SD 8.4 cm²/s in Fu vs 4.0, SD 1.6 cm²/s in FRb). Complete assistance of the cobot significantly reduced interindividual variability of all center of pressure parameters. In FRb modality, as compared with all other conditions, removing the weight of the object during loading or unloading phases caused a significant decrease in all parameter values.

**Conclusions:**

Increased cobot assistance significantly enhances postural stability and reduces biomechanical load on workers during simulated tasks. Full assistance from cobots, in particular, minimizes postural displacements, indicating more consistent postural control improvements across individuals. However, high levels of cobot intervention also reduced the natural variation in how people balanced themselves. This could potentially lead to discomfort in the long run. Midlevel cobot assistance modalities can thus be considered as a good compromise in reducing biomechanical risks associated with postural stability at the same time granting a satisfactory level of user control.

## Introduction

Industrial manufacturing is transitioning from well-established production procedures toward more flexible and intelligent manufacturing systems (Industry 4.0, as initially described by Kagermann et al [1]). This evolution aims to develop innovative, sustainable solutions that create new business models, improve working conditions, increase plant productivity, and enhance product quality [2]. Robotics plays a crucial role in this context, with industrial robots being widely adopted due to their ability to relieve humans from repetitive, unhealthy, or dangerous tasks [3]. The field of robotics has evolved to not only allow humans to share the same workspace with robots but also use them as assistants, thereby enhancing human-robot collaboration (HRC) [4].

Collaborative robots (cobots) are at the forefront of this revolution, offering increased productivity, flexibility, versatility, and safety compared with traditional industrial robots. Cobots are designed to work alongside humans, sharing the same workspace without the need for safety cages and providing greater mobility and flexibility [5]. The integration of cobots in industrial environments has been shown to significantly enhance operational efficiency [6] and worker satisfaction by enabling safer and more ergonomic working conditions [7,8]. However, the integration of cobots impacts various human factors, which include psychological aspects [9], emotions [10], and biomechanical effects [11]. Consequently, it is important to consider both cognitive ergonomics and biomechanical safety to optimize the overall effectiveness and well-being of human workers [12]. For instance, Gualtieri et al [13] have investigated the cognitive elements involved in human interaction with cobots, revealing significant insights into how mental workload and task complexity can affect worker performance and satisfaction [14]. Similarly, improper collaboration with cobots can expose workers to biomechanical risks, even though these robots are intended to alleviate heavy and tedious tasks. Previous research studies [15,16] emphasized the importance of ergonomic considerations in HRC to prevent musculoskeletal disorders (MSDs) among workers.

Thus, the right choice of cobot collaboration modality can reduce biomechanical overload on workers, ensuring their safety.

Recent advancements in sensor technology and data analytics have enabled detailed assessments of human biomechanics during HRC [17,18]. Studies have shown that real-time monitoring and analysis of physiological data can provide valuable insights into the physical strain experienced by workers and help in optimizing collaborative processes [19]. For example, the use of wearable sensors and motion capture systems could effectively measure and analyze the biomechanical load on workers during different collaborative tasks with robots [20]. Other recent studies investigated the effects of different HRC modalities on physiological human parameters such as trunk oscillations [21] and muscle coactivation [22,23]. Such studies can also be profitably taken into consideration for the optimization of workplace design and task allocation.

When work tasks are performed in quasi-static conditions, direct measures of posture could reveal insights into the stability and balance of workers and can thus be used to improve ergonomics during HRC, as they can help identify postural adjustments and biomechanical loads, providing a more comprehensive understanding of HRC ergonomic impact [24].

Following this principle, this study will evaluate and analyze posturography data to assess the biomechanical risks in HRC working contexts where cobots are used in different modalities. Through this evaluation, the study seeks to determine whether cobot assistance can effectively reduce biomechanical risks: by examining how different collaborative modalities influence workers’ stability and balance, thus providing insights into the effects of cobot assistance on postural control, as this latter one indirectly reflects safety, efficiency, and ergonomic well-being of human workers in collaborative environments.

## Methods

### Experimental Setup

#### Overview

**Figure 1.**
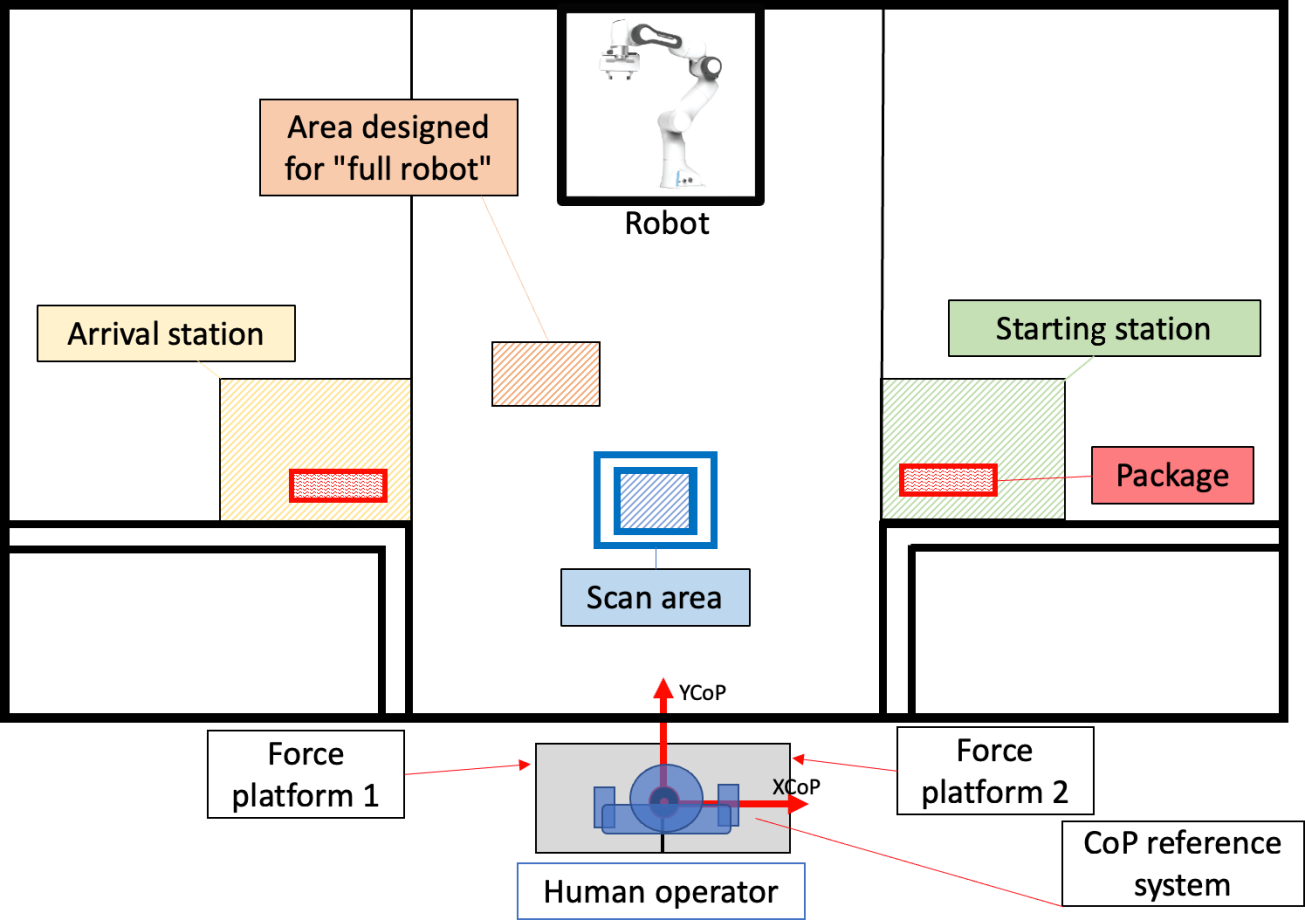
Experimental setup dimensioning for the simulation of load-moving activities on a workbench. CoP: center of pressure.

The setup was designed to compare a simulated work activity performed with and without cobot assistance. A specific workbench was created by positioning the cobot on one of the longer sides of a 2 × 1 m table, with the operator standing upright on the opposite side (Figure 1). The operator’s standing position was fixed relative to the edge of the table, defined by the position of his or her feet and the projection onto the ground of the long side of the table. A starting station (ie, the initial position to the right of the operator) and an arrival station (ie, the final position to the left) were defined, each 1m apart from the other and 30 cm away from the operator in the direction of the cobot. A scanning area was defined in the center of the table in front of the operator. This workbench arrangement allowed the operator to perform all the activities (or part of them) independently, while the cobot could collaborate on some or none of the tasks without interfering with the operator’s movements. This configuration was defined to minimize possible hindering of the operator’s movements.

The activities involved the handling and relocating of rectangular packages, each measuring 14 × 12 × 5 cm and weighing 1 kg, bearing a QR code on one side.

The tests were conducted with a group of 14 male volunteers aged between 25 and 48 years (weight: 79, SD 7 kg; height: 180, SD 5 cm). All volunteers were healthy and had no evident or declared mobility issues. Moreover, given the design of the setup, only right-handed subjects have been involved in the tests. Participants were instructed to (1) grasp the packages from the starting station, (2) identify the QR code and scan it (ie, facing the QR code to the scan area) in the central area, and then (3) place the package in the arrival station. Different levels of collaboration were implemented by removing the first part of these activities (ie, grasping the package and bringing it to the center of the table) when in partial assistance, and removing also the last part (ie, bringing the package to the arrival station) when in full cobot assistance. Each participant was asked to perform the task as naturally as possible. This task was designed to simulate the work activity of a cashier in a supermarket, a highly relevant example in terms of strenuous activity [25], where an increase in the level of cobot assistance is hypothesized to reduce the associated biomechanical risk.

In the test, each participant performed consecutive repetitions of the same task, for a 5-minute duration, with different levels of cobot assistance.

The frequency of processing (packages per minute [PPM]) to be handled was not defined a priori, and participants were allowed to choose their own pace in performing all the activities. When collaborating with the cobot, the PPM frequency was however influenced by the cobot’s package handling activity, as described below.

Each participant was asked to perform the described activity under 4 different collaboration modalities, performed in random order, with a 5-minute break between each test.

#### Full (Fu)

In this baseline scenario, the operator performs the tasks independently, without cobot collaboration. The operator picks up the package from the starting station with the right hand, examines it with both hands to locate the QR code, brings it close to the scanner simulation area, and finally places the package in the arrival station with the left hand. The processing rate was freely self-selected by each participant.

#### Half Robot Touch (HRT)

In this condition, the cobot task is to pick up the package from the starting station and wait for the operator to extend their right arm toward the same area, and to touch it to start. When touched, the cobot moves bringing the package in front of the operator who grabs and examines it with both hands to locate the QR code. The operator then brings it close to the scanner simulation area and finally places the package in the arrival station with the left hand. The task is kinematically like the “Full” case, but there is no lifting of the package from the starting station toward the scanning area. Moreover, the operator drives the activation of the cobot with a touch, and therefore the number of PPM is decided by everyone, taking into account the cobot speed.

#### Half Robot (HRb)

In this modality, the cobot’s task is to pick up the package from the starting station and autonomously transport it in front of the operator, who grabs and examines it with both hands to locate the QR code. The operator then brings it close to the scanner simulation area and finally places the package in the arrival station with the left hand. The operator is not required to initially reach the right arm toward the starting station to ask the cobot for help, so the task is different in terms of kinematic features compared with the previous cases. Packages are moved continuously by the cobot while the operator is performing the required tasks, therefore, the cobot determines the number of PPM.

#### Full Robot (FRb)

In this condition, the cobot’s task is to pick up the package from the starting station and autonomously transport it continuously in front of the operator, who grabs and examines it with both hands to locate the QR code. The operator then brings it close to the scanner simulation area and finally places the package in a closer area with respect to the arrival station (Figure 1). Meanwhile, the cobot picks up the package left by the operator and takes it to the arrival station. The cobot intervenes significantly, fully collaborating in the activity. However, the time taken by the cobot to move the packages always exceeds the time that the operator needs to perform the activity. Therefore, the operator must wait for the cobot, and the motor activity is discontinuous, with the number of PPM defined by the cobot kinematics.

### Ethical Considerations

The experimental protocol was approved by the Commissione Etica dell’Università degli Studi Roma Tre (BRIC2019-BRISK approval r.01-10/06/2021). Informed consent was obtained from all participants prior to their inclusion in the study, and they were informed of their right to withdraw at any time without consequences. All data collected were anonymized to protect participants' privacy and confidentiality. No personally identifiable information was retained, and strict security measures were implemented to safeguard the data. Participants did not receive any financial or material compensation for their involvement in this study.

### Data Acquisition and Processing

To assess the dynamic interaction of the operator with the ground, two 6-component force platforms (BTS P-6000) were positioned under the participants’ feet. These platforms allow for measuring the ground reaction of each participant’s lower limbs (force and moment components), from which the center of pressure (CoP) position of each foot is extracted during the execution of performed tasks. Data were acquired from force platforms using a sampling frequency of 500Hz (fixed by the HW system). An optoelectronic system (SMART DX 6000, 8 infrared cameras, 2 Mpixel@300 Hz) was employed to segment and identify each task repetition, and each phase of the repetition. Data from the optoelectronic system were acquired using a sampling frequency of 250Hz. Force platforms data were undersampled at 250 Hz to synchronize those with the optoelectronic system ones.

### Dynamic Parameters Extraction

Trajectories of the CoP for each of the lower limbs (ie, CoP-right and CoP-left) during the execution of each task were extracted from the force plates data [26]. The total CoP is obtained as the weighted average between CoP-right and CoP-left and its displacement is defined by both the medial-lateral and the anterior-posterior coordinates with respect to a reference system centered between the 2 force plates (Figure 1).

Four CoP-derived parameters were calculated to evaluate the postural stability of the operator and quantify alterations in balance: for each of the collaboration modalities, the mean distance (MDIST), mean velocity (MVELO), 95% confidence ellipse area (AREA-CE), and sway area (AREA-SW) were calculated, according to the definitions reported in [27], for each cycle of the task repetition. For each parameter, mean values (as a measure of central tendency) and SD across repetitions (as a measure of intraindividual variability) were also calculated and underwent inferential statistics.

Cycles were identified using data extracted from motion capture data, which were synchronized with force plate data: in particular, the time location of the maximum excursion to the left in the medio-lateral direction of the marker placed on the right wrist was used to segment each cycle, as it corresponds to the instant when each repetition finishes.

### Statistical Analysis

For each CoP-derived parameter thus obtained, the normality of the data, using the Shapiro-Wilk test, was assessed. When all distributions were normal, the homogeneity of the variances was checked with the Leneve test. If these conditions were not satisfied, the Kruskal-Wallis nonparametric test was applied to examine the effect of the collaboration modality. When significant, a post hoc test with a Bonferroni correction was conducted to investigate the differences among the 4 examined collaboration modalities. The α level was set to .05 and .01 (denoted by * and **, respectively).

## Results

### Rate of Package Processing

The average rate of package processing chosen by the operators in the absence of cobot assistance (Fu) was 15 (IQR 14-16) PPM. The different cobot collaboration modalities yielded median rates of 8 (IQR 7-8.5) for PPM for half robot touch (HRT), 12 (IQR 11.5-13) PPM for half robot (HRb), and 4 (IQR 3.9-4.1) PPM for full robot (FRb).

### CoP Trajectories

In the following, the different trajectories of the CoP, for all 14 participants and for the 4 different collaboration modalities (ie, full [Fu], HRB, HRb, and FRb) are shown, with Xcop and Ycop denoting respectively the medium-lateral and the antero-posterior coordinate.

**Figure 2.**
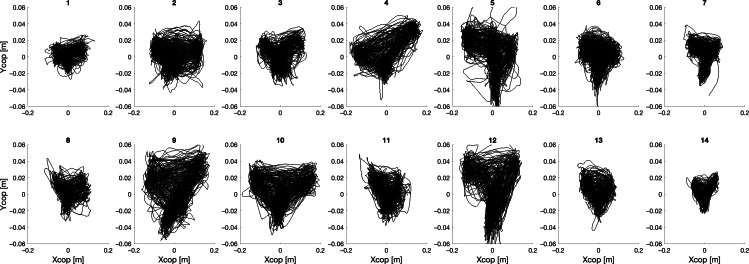
Center of pressure (cop) trajectories for each participant during the full collaboration modality.

Considering the Fu modality (Figure 2), observed CoP paths can be classified into 2 main groups: participants (1,2,3,6,7,8,11,13,14) displayed a compact size of the CoP path, spread in both anterior-posterior (AP) and medial-lateral (ML) directions, while participants (4,5,9,10,12) drew wider areas, with a remarkable trajectory from the central backward position to the right forward position. However, all the CoP paths present quite diverse behavior, with no visible common aspects. Negative values for Ycop denote backward displacements.

**Figure 3.**
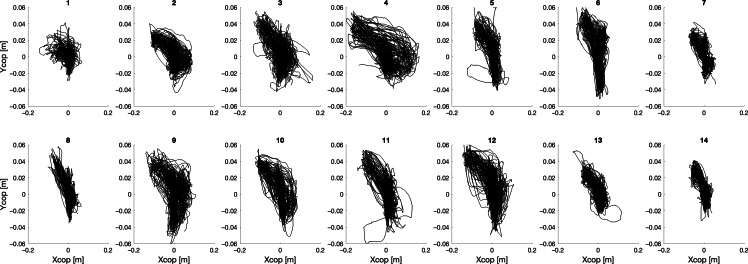
Center of pressure (cop) trajectories for each participant during the half robot touch collaboration modality.

Considering the HRT collaboration modality (Figure 3), CoP path can be classified into 2 main groups also in this case: participants (1,2,7,8,13,14) maintained a compact size of the CoP path, more spread along the AP direction, while participants (3,4,5,6,9,10,11,12) drew a wider area of the CoP path. In this case, all CoP trajectories denoted a bias from the central backward position to the left forward position. Displayed CoP paths still exhibit rather diverse behavior, but with more commonalities as compared with Fu modality.

**Figure 4.**
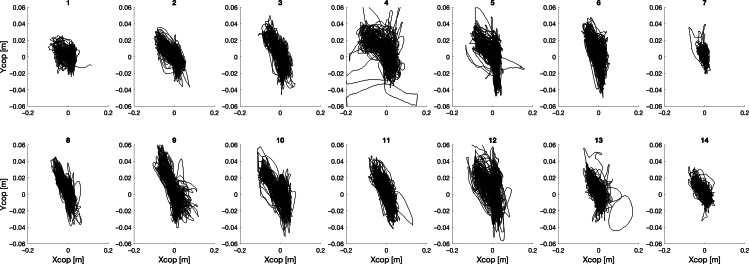
Center of pressure (cop) trajectories for each participant under the half robot collaboration modality.

For the HRb collaboration modality (Figure 4), also in this case CoP path can be classified into 2 main groups: for participants (1,2,7,13,14) CoP path was maintained compact, differently from participants (3,4,5,6,8,9,10,11,12), especially considering the AP coordinate. Common behavior of CoP excursion across subjects was instead present along the ML coordinate, while the CoP bias from the central backward position to the left forward position observed in the HRT case is less evident.

**Figure 5.**
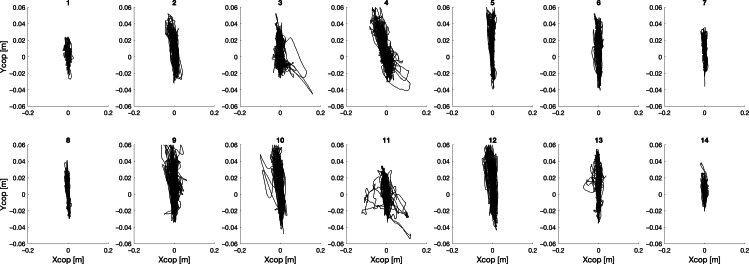
Center of pressure (cop) trajectories for each participant during the full robot collaboration modality.

In the FRb collaboration modality (Figure 5), commonalities of CoP behavior are more relevant. While some participants still display lower excursions along the AP direction, the characteristics of CoP trajectories are mostly similar, with no bias both in the right and left forward positions. The amount of excursion in the ML direction is similar across participants.

### Normality Tests

The Shapiro-Wilk test indicated that some conditions of the parameters did not follow a normal distribution. For the MDIST values, a *P* value <.05, indicating rejection of the null hypothesis of normal distribution, was observed for the Fu condition (*P*=.048). In contrast, a normal distribution (*P* value >.05) was found for all the other conditions (HRT: *P*=.44; HRb: *P*=.57; FRb: *P*=.60).

Similar results were obtained for both the AREA-CE and AREA-SW parameters. The Fu condition showed a nonnormal distribution (*P*=.02 for AREA-CE and *P*=.04 for AREA-SW), while all other conditions were normally distributed (for AREA-CE: HRT, *P*=.18; HRb, *P*=.25; FRb, *P*=.33; for AREA-SW: HRT, *P*=.08; HRb, *P*=.86; FRb, *P*=.25).

Since at least 1 of the distributions for each parameter was not normal, nonparametric statistical analyses were applied.

For the MVELO parameter, data were normally distributed across all conditions. However, Levene test revealed that the variances among the different conditions were not homogeneous (*P* value <.05). Consequently, a nonparametric test was also used for this parameter.

### CoP-Derived Parameters

Descriptive statistics at the individual level, and descriptive and inferential statistics at the group level for MDIST, MVELO, AREA-CE, and AREA-SW, as obtained across the 4 collaboration modalities, are given in the following figures.

**Figure 6.**
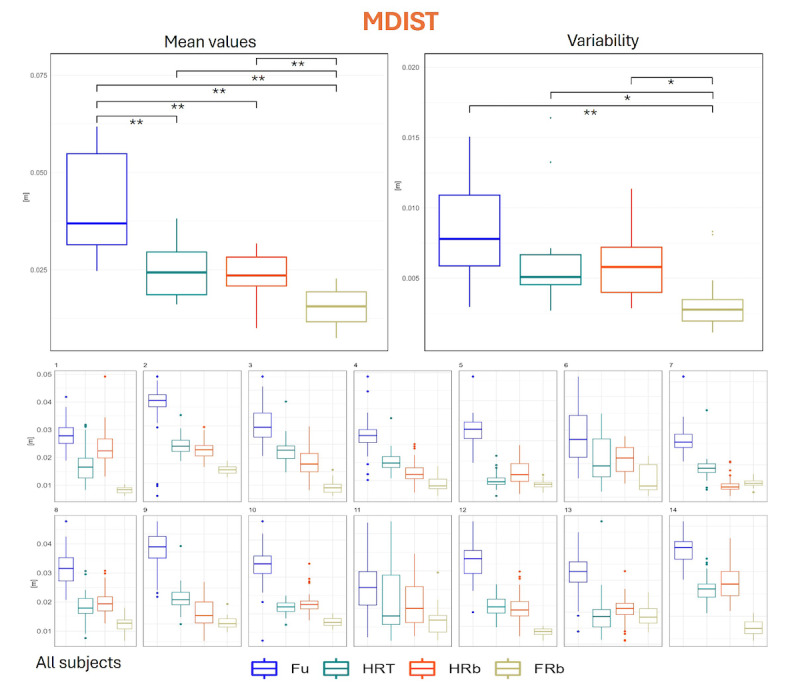
Distribution of MDIST values. Upper panel, left: group mean values across the 4 collaboration modalities; upper panel, right: distribution of intraindividual variability (SD across repetitions); lower panels: distribution of values for each participant. * denotes *P*<.05 as obtained from inferential statistics; ** denotes *P*<.01 as obtained from inferential statistics. FRb: full robot; Fu: full; HRb: half robot; HRT: half robot touch; MDIST: mean distance.

MDIST, an overall measure of excursion of the CoP from its equilibrium position, was clearly and significantly higher in Fu condition (4.21, SD 1.30 cm) than in all other conditions, and this was confirmed also at the individual level for almost every participant. In addition, as shown in Figure 6, FRb condition caused significantly lower values (1.55, SD 0.50 cm) than both HRT (2.50, SD 0.67 cm) and HRb (2.38, SD 0.59 cm), and this appeared also in all individuals but 2. No significant difference was observed between HRT and HRb conditions, as the result of different behavior across participants. Regarding intraindividual variability, we observed a reduced variability of MDIST across most participants in FRb condition (0.34, SD 0.22 cm) as compared with HRb (0.59, SD 0.23 cm), HRT (0.65, SD 0.37 cm), and Fu (0.85, SD 0.36 cm).

**Figure 7.**
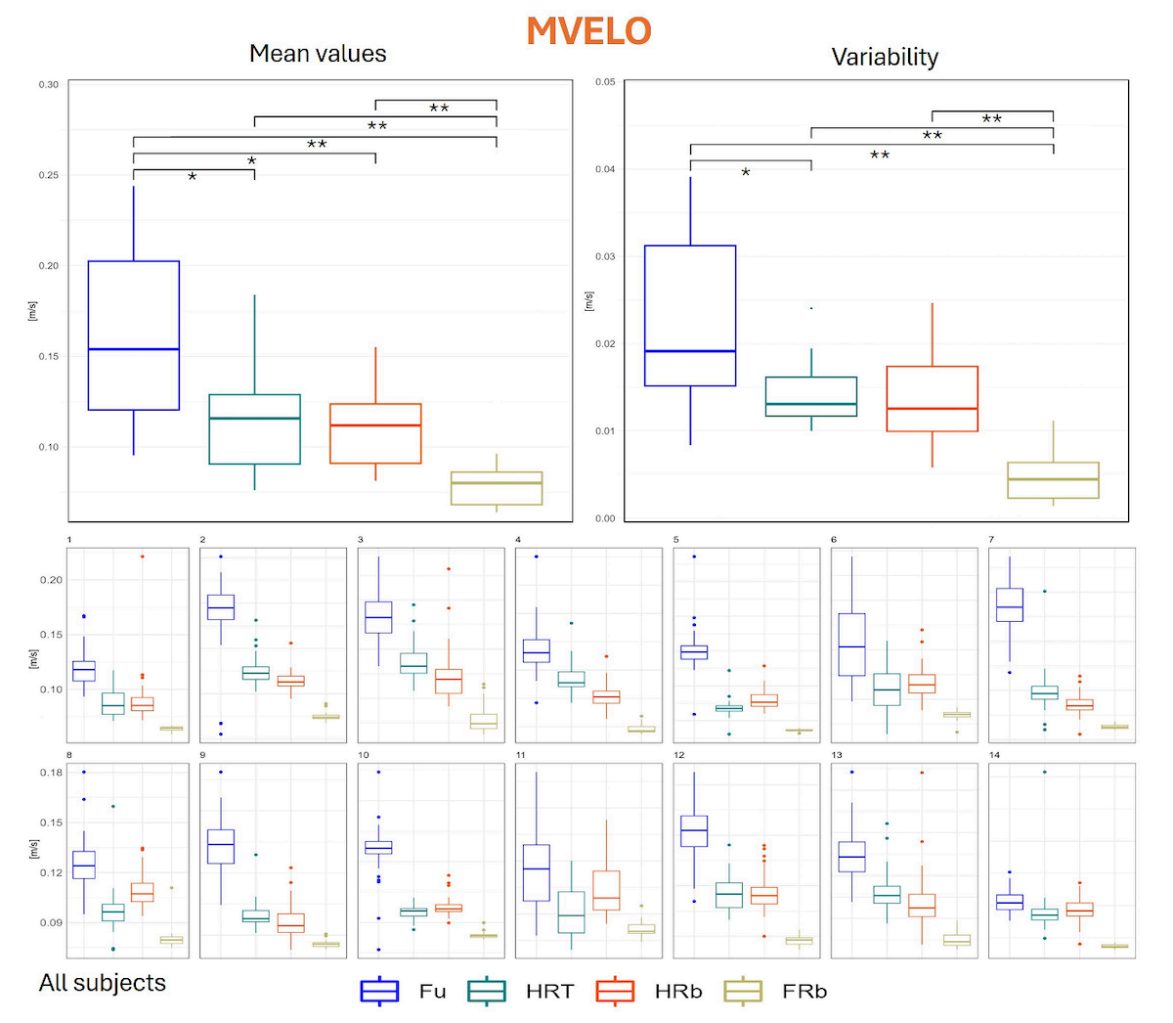
Distribution of MVELO values. Upper panel, left: group mean values across the 4 collaboration modalities; upper panel, right: distribution of intraindividual variability (SD across repetitions); lower panels: distribution of values for each participant. * denotes *P*<.05 as obtained from inferential statistics; ** denotes *P*<.01 as obtained from inferential statistics. FRb: full robot; Fu: full; HRb: half robot; HRT: half robot touch; MVELO: mean velocity.

MVELO, an indicator of the amount of movement described by the CoP trajectory, in general confirms what already reported for MDIST: Fu condition determined higher values (16.3, SD 5.2 cm/s) than all other collaboration conditions, and in FRb, MVELO was significantly lower (7.9, SD 1.1 cm/s) than in all other conditions (11.1, SD 2.2 cm/s for HRb; 11.3, SD 2.9 cm/s for HRT). As depicted in Figure 7, these figures were confirmed also at the individual level for all but 2 participants. In terms of variability, while we observed that it was mostly reduced in the FRb condition (0.5, SD 0.3 cm/s) as compared with all the other conditions (2.3, SD 1.0 cm/s for Fu; 1.4, SD 0.5 cm/s for HRb), a significant decrease of the variability was present also when moving from Fu to HRT (1.4, SD 0.4 cm/s). Other variations were not significant at the group level.

**Figure 8.**
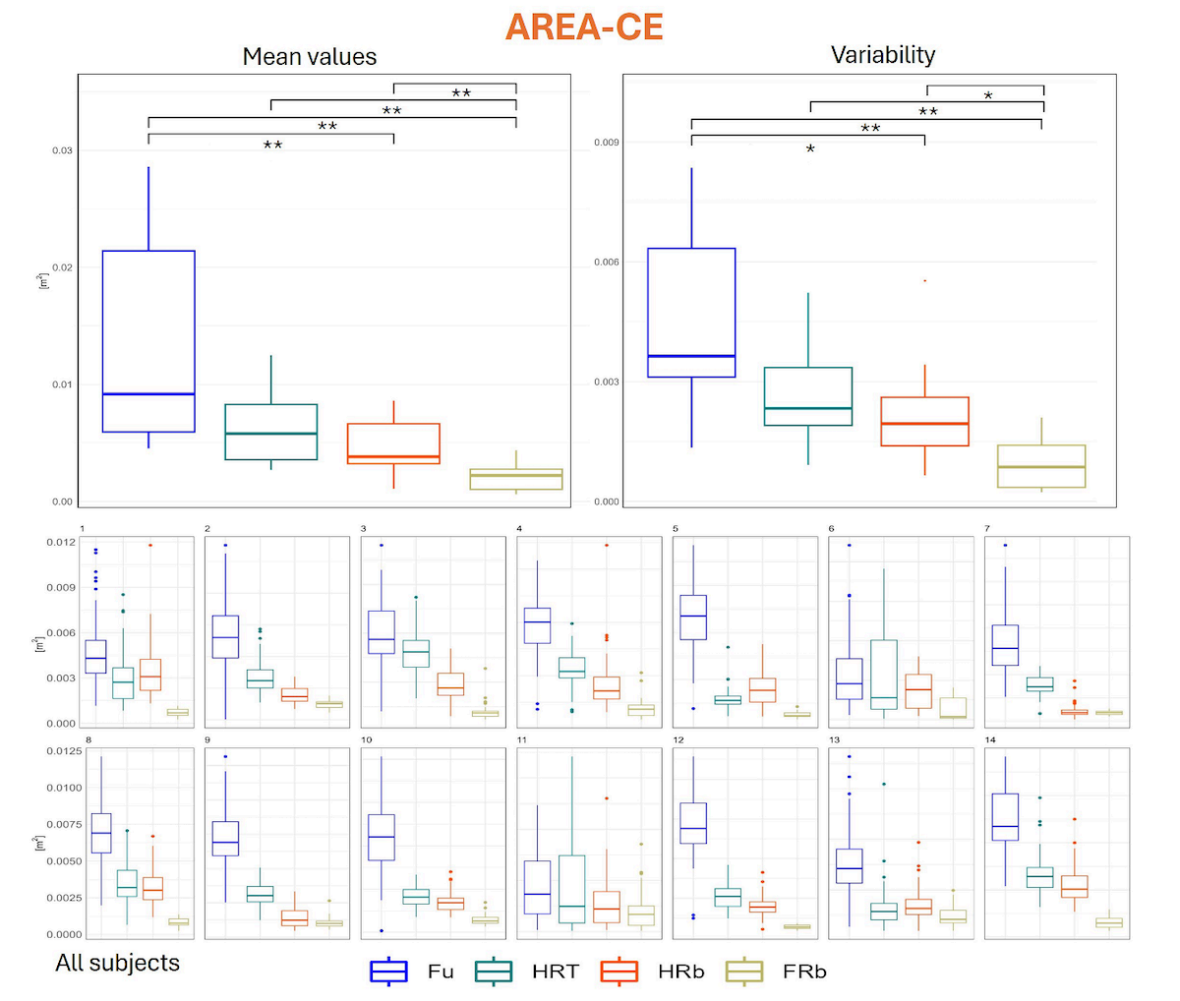
Distribution of AREA-CE values. Upper panel, left: group mean values across the 4 collaboration modalities; upper panel, right: distribution of intraindividual variability (SD across repetitions); lower panels: distribution of values for each participant. * denotes *P*<.05 as obtained from inferential statistics; ** denotes *P*<.01 as obtained from inferential statistics. AREA-CE: confidence ellipse area; FRb: full robot; Fu: full; HRb: half robot; HRT: half robot touch.

AREA-CE, which quantifies the planar extent of coverage of the CoP, displayed a behavior similar to what was seen for MVELO: values resulted significantly higher in Fu (134, SD 91 cm²), and lower in FRb (22, SD 12 cm²) than in all the other conditions (64, SD 32 cm² for HRT; 47, SD 24 cm² for HRb), respectively (Figure 8). Again, FRb intraindividual variability was lower in FRb (10, SD 7 cm²) than in all other conditions, and we saw a reduced variability when passing from Fu (45, SD 23 cm²) to HRb (21, SD 12 cm²).

**Figure 9.**
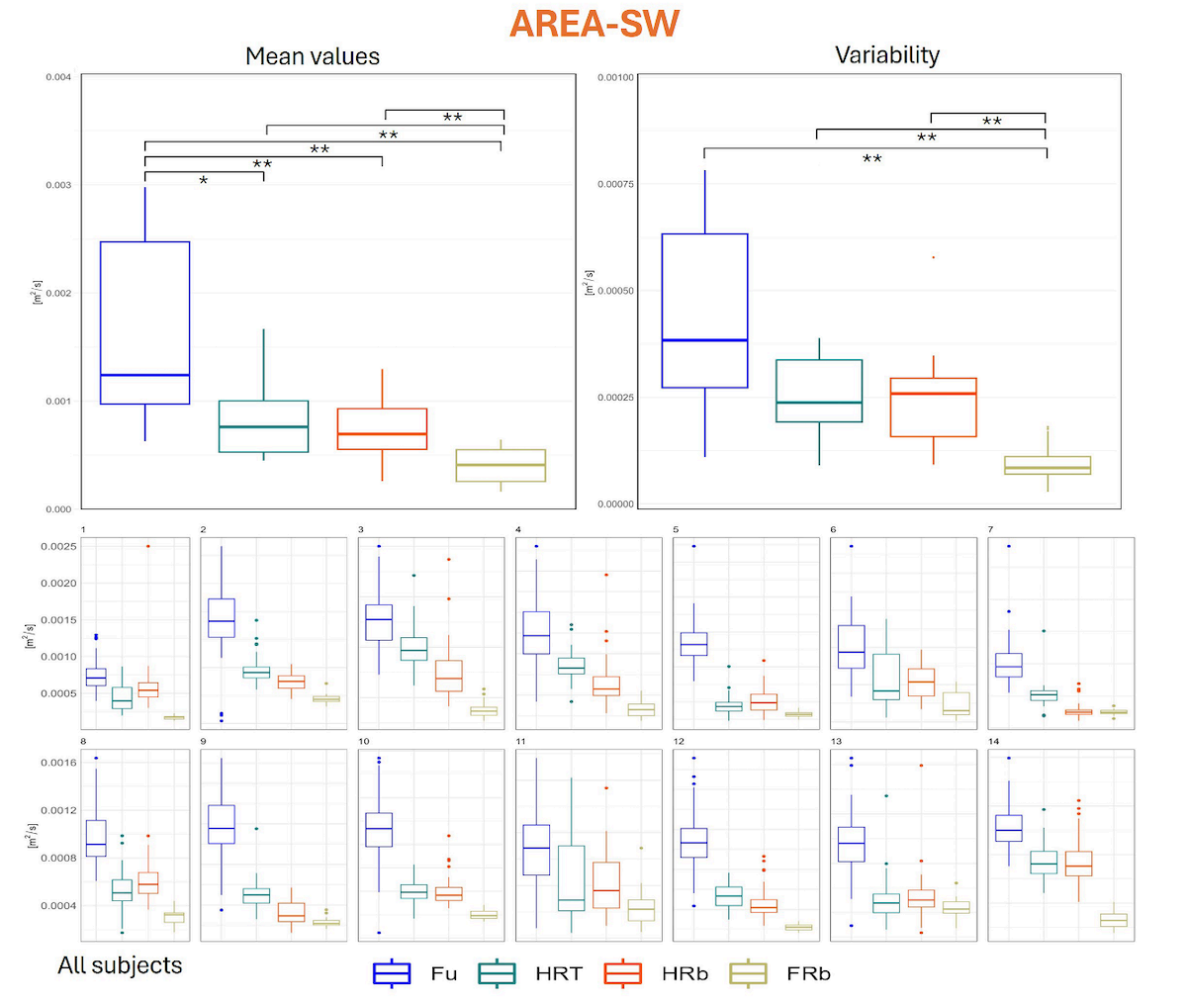
Distribution of AREA-SW values. Upper panel, left: group mean values across the 4 collaboration modalities; upper panel, right: distribution of intraindividual variability (SD across repetitions); lower panels: distribution of values for each participant. * denotes *P*<.05 as obtained from inferential statistics; ** denotes *P*<.01 as obtained from inferential statistics. AREA-SW: sway area; FRb: full robot; Fu: full; HRb: half robot; HRT: half robot touch.

Finally, AREA-SW, a planar measure that combines CoP extension and velocity, displayed behavior similar to what obtained with MDIST: Fu caused significantly higher AREA-SW values (16.2, SD 8.4 cm²/s) than all the other conditions (8.4, SD 3.7 cm²/s and 7.3, SD 2.8 cm²/s for HRT and HRb, respectively), and again FRb brought to values lower (4.0, SD 1.6 cm²/s) than all the other collaboration modalities (Figure 9). Both these results were reflected at the individual level for all participants but one. Intraindividual variability of AREA-SW was significantly lower in FRb (0.9, SD 0.4 cm²/s) than in all other conditions (4.4, SD 2.2 cm²/s; 2.5, SD 0.8 cm²/s; and 2.5, SD 1.2 cm²/s for Fu, HRT, and HRb, respectively).

## Discussion

### Principal Findings

The analysis of the CoP trajectories shows that considering data obtained in tests going from the absence of cobot intervention (Fu) to the ones with maximum cobot assistance (FRb), the area of the CoP trajectory significantly decreases, thus possibly indicating a lower amount of oscillations of the body when the cobot intervenes.

As a general consideration, CoP trajectories, when no action of the cobot is present, tend to show greater intensity toward the loading area, indicating a higher amount of sway. This suggests that lifting affects balance control strategies. On the other hand, in both tests involving partial cooperation with the cobot, the CoP draws trajectories mainly toward the arrival station, even in the HRT modality, where the participant’s arm moves toward the starting station direction to ask the cobot for help. This aspect suggests that the movement of the arm while carrying the load notably influences the CoP trajectory, while the arm movement, required to ask the cobot for help occurring in the HRT condition, does not produce a significant influence on the CoP trajectory, as compared with the modality where the cobot independently carries the load to the scanning zone (ie, HRb collaboration modality).

Regarding the detailed analysis of the specific CoP trajectories of all subjects and for all test types, results show that during tasks performed alone, CoP paths can be classified into two types: (1) concentrated with symmetrical movements toward both the starting and arrival stations (right and left), and (2) more scattered with a tendency to oscillate between the central rest zone and the arrival station (left). The variability of CoP trajectories across individuals may confirm the adoption of different independent control strategies, as also identified by the 2 types listed before. When instead maximum assistance by the cobot is present, areas drawn by CoP are smaller, and with a reduced interindividual variability. This homogenization effect can be explained by the fact that the cobot sets the activity’s pace, causing the subject to adopt a more uniform behavior and timing. This is both apparent at the individual level (ie, each subject tends to maintain a more uniform CoP trajectory across repetitions), and at the group level.

Results coming from the qualitative description of CoP trajectories were also confirmed by statistical analysis of CoP-derived parameters: specifically, CoP tends to travel more (MVELO and, to a certain extent, AREA-SW) and further (MDIST and AREA-CE) if the level of intervention from the cobot increases. In general, smaller excursions can be associated with improved balance: this means a higher tendency to remain in a smaller (and generally more stable) area during task repetitions. A possible interpretation is that while the cobot influences task execution, it also makes the operator move less with reduced postural oscillation. This aspect is relevant as an increased excursion from the equilibrium position and increased movement of the CoP are associated with an increased risk of falling [28], and incorrect postures in the working scenario are agreed to lead to the development of MSDs [29]. As seen from this perspective only, the modality of full assistance by the cobot brings to the lowest risk, as it clearly leads to minimal excursions and movements of the body.

However, we also observed a marked reduction of intraindividual variability of these parameters when full assistance from the cobot is present: one hypothesis for this evidence is that the presence of the cobot reduces autonomy in task execution and may thus impose a control strategy which is not necessarily beneficial (or chosen) by the operator. If a reduction of variability of such parameters from the baseline condition may be seen as an indicator of long-term discomfort, this needs to be appropriately considered when designing the most beneficial collaboration modalities. In this regard, intermediate collaboration modalities seem to incorporate a tradeoff between the advantages in the carrying task compensation given by the cobot on the load and the possibility of granting autonomy to the operator during the task execution.

Regarding the 2 collaboration modalities that maintain a material operator intervention, statistical analysis at the group level was rather inconclusive: in excursion-based CoP parameters, no difference was found, and this is counterintuitive, considering that one of the modalities required participants to raise the arm and kinematically reach out to ask for assistance. This was not reflected in most CoP-derived parameters, and the possible explanation for this absence of differences may be ascribed to the inherent nature of the CoP variable: it is only indirectly associated with whole body movement: coming from the ground reaction forces, it is a measure of where the foot pressure is concentrated [30]. In static conditions, CoP basically tracks the body barycenter projection to the ground (and dynamically overtakes it in specific conditions) to counteract balance disequilibrium. According to the inverted pendulum hypothesis, the body barycenter projection is thus a filtered version of the CoP trajectory. However, with self-initiated body part movements, the central nervous system is able to predict future whole-body excursions [31], and the position of the CoP may not necessarily reflect the excursions of the body barycenter. If this were the case, activation of the relevant muscles (typically, tibialis anterior) would appear in anticipation of the upper limb movement.

Regarding the inconclusive results appearing between the 2 modalities where collaboration was midlevel (ie, HRb and HRT), we tend to ascribe this process to a rather diverse behavior observed at the individual level: decreases of measures of CoP excursion and movement were in some individuals clear when decreasing the amount of user intervention (ie, passing from HRT to HRb), however this effect disappears in a nonnegligible share of individuals; as it was apparent from the qualitative description of CoP trajectories, 2 different group behaviors seemed to emerge: in one group, CoP is maintained compact despite the lesser level of cobot intervention as compared with FRb, while the second group displays higher amounts of excursion and movement. We were not able to ascribe this phenomenon to specific individual characteristics; whole body kinematics and electromyography activity analysis may help shed light on this, in particular, to check if this evidence might be associated with task role-taking, which may be a key factor in HRC activities [32].

Reduced postural variability, as evidenced by the decreased CoP trajectory area and reduced inter and intraindividual differences under maximum cobot assistance, can have significant long-term implications for musculoskeletal health. A prolonged state of limited variability in posture and movement, while potentially stabilizing in the short term, may contribute to the development of MSDs over time. This is because variability in postural strategies allows the redistribution of mechanical loads across different tissues and joints, reducing the risk of overuse injuries or chronic strain on specific musculoskeletal structures. Conversely, homogenized and repetitive postures imposed by external systems, such as cobots, may lead to localized fatigue, reduced muscular engagement, and impaired adaptability of the neuromuscular system. Consequently, these factors can heighten the susceptibility to cumulative trauma disorders, particularly in dynamic occupational scenarios. These findings highlight the importance of designing collaborative robotic systems that balance task support with opportunities for natural and varied movement patterns to mitigate potential long-term health risks for operators.

The implications of these findings for industrial settings may be interesting. The integration of cobots can significantly enhance worker safety and productivity by reducing physical strain and improving postural control. By enabling safer and more ergonomic working conditions, cobots can play a crucial role in creating healthier work environments, reducing the incidence of work-related MSDs, and improving worker satisfaction.

In general, referring to the obtained results, it must be remarked that the tasks analyzed were simulated rather than conducted in real-world contexts, so further analysis could be conducted to generalize the findings. However, the tests conducted in this study, even if simulating a cashier task, represent real work tasks themselves, and this supports the hypothesis that similar results can be obtained in the real world. Additionally, the sample consisted exclusively of young male participants, so a direct validation on female subjects and older ones was not conducted. Rather than a limit, this might be seen as a validation for the examined population, although it can be extended to other worker categories.

### Conclusions

This study investigated the biomechanical impact of various levels of HRC on postural control during working tasks while standing, providing substantial insights into the benefits of cobot assistance for workers’ safety and ergonomics. Through an analysis of CoP trajectories and related parameters, it was found that higher levels of cobot collaboration, particularly in the HRT, HRb, and FRb modalities, significantly improved postural stability and reduced biomechanical load on workers. These findings suggest a decreased risk of MSDs, as evidenced by lower values in CoP-derived parameters when cobot assistance increases. The study also highlighted that the intraindividual variability of CoP parameters diminished when levels of cobot involvement were increased: this indicates on one side that cobot integration promotes more uniform ergonomic benefits across different individuals, but at the same time it may tend to impose a similar behavior in balance control which can lead subjects to adopt less ecological strategies.

Moreover, while participants can set autonomously their repetition pace, HRC activities will determine an increased homogenization of repetition rhythm (or parts of it) across workers, and this may potentially lead to decrease ergonomics, as subjects can feel forced to perform the required tasks based on timings dictated by HRC. Intermediate collaboration modalities, such as HRT, can provide both advantages in terms of reduction of CoP-derived parameters—that can be indirectly associated with a reduced biomechanical risk—with the possibility of selecting a more comfortable rhythm in executing tasks.

However, further research is needed to explore the long-term effects of cobot assistance on worker health and productivity. In particular, future studies should include other sensing technologies, able to capture the neuromechanics of HRC. In addition, application to diverse populations and real-world industrial settings to generalize the results and provide a more comprehensive understanding of the benefits and potential challenges of cobot integration. Additionally, exploring the psychological and social impacts of working with cobots on employees would provide a holistic view of the implications of collaborating scenarios in industrial environments: if properly integrated into working environments cobots can potentially revolutionize industrial work by enhancing safety, efficiency, and ergonomic well-being, facilitating the development of more intelligent and flexible workrooms and working conditions.

## Abbreviations

AP: anterior-posterior
AREA-CE: confidence ellipse area
AREA-SW: sway area
cobot: collaborative robot
CoP: center of pressure
FRb: full robot
Fu: full
HRb: half robot
HRC: human-robot collaboration
HRT: half robot touch
MDIST: mean distance
ML: medial-lateral
MSD: musculoskeletal disorder
MVELO: mean velocity
PPM: packages per minute
